# Bridging the Gap – Physical Health Management by Mental Health Nurses in Pendleview: A Quality Improvement Project

**DOI:** 10.1192/bjo.2025.10333

**Published:** 2025-06-20

**Authors:** Faisal Alam, Celina Mitala, Aarthi Krishnan, Ushbah Ali, Abdalla Salih

**Affiliations:** LSCFT NHS Foundation Trust, Blackburn, United Kingdom

## Abstract

**Aims:** To reinforce nurses’ initial physical health management knowledge on Darwen and Calder wards at Pendleview unit (LSCFT). This quality improvement project attempted to bridge the gap (of physical health knowledge) amongst nursing staff by providing a short teaching course of 3 topics to mental health nurses on Darwen and Calder ward at Pendleview mental health unit, Royal Blackburn Hospital.

**Methods:** The quality improvement project was conducted using the PDSA (plan, do, study, act) cycle methodology. The sample included 20 mental health nurses across Darwen and Calder wards in Pendleview unit. Three teaching sessions were delivered to nursing staff by doctors on both Darwen and Calder wards (6 in total) covering blood sugar monitoring, EWS and escalation and pain management. Quantitative and qualitative data was collected via pre- and post-teaching feedback forms, assessing nurses’ confidence and knowledge in managing physical health conditions. Confidence and knowledge were both scored on Likert scales numbered from 1–5.

**Results:** 85% of nurses (17 of 20) stated they had not received training on the teaching topics before starting work Data across the three teaching sessions revealed the following;

Blood sugar monitoring (n=8): Mean confidence (1 – not at all confident, 5 – confident) increased from 2.75 95% CI [1.85, 3.65] to 4.75 95% CI [4.45, 5.05] out of 5. Mean knowledge (1 – very poor, 5 – extremely good) increased from 2.75 95% CI [2.292, 3.208] to 4.75 95% CI [4.45, 5.05] out of 5.

EWS and escalation (n=6): Mean confidence increased from 3.5 95% CI [2.493, 4.507] to 4.3 95% CI [3.704, 4.896] out of 5. Mean knowledge increased from 3.5 95% CI [3.1, 3.9] to 4.5 95% CI [4.1, 4.9] out of 5.

Pain management (n=6): Mean confidence increased from 4.33 95% CI [3.953, 4.707] to 4.83 95% CI [4.532, 5.128] out of 5. Mean knowledge increased from 3.5 95% CI [3.1, 3.9] to 4.67 95% CI [4.293, 5.047] out of 5.

**Conclusion:** Physical health management teaching to mental health nursing staff has shown to increase nurses’ confidence and knowledge in physical health. Providing physical health management teaching trust wide can help to eliminate knowledge gaps among the nursing staff, irrespective of their prior knowledge. Flow charts, posters, and providing regular physical health teaching and training to nurses during induction and beyond can all aid to empower nursing staff. A further QI cycle could be explored, looking into new teaching content after determining any additional gaps in physical health knowledge of the nursing staff.

